# Retroperitoneoscopic surgical resection of a crossed ectopic non-functioning multicystic kidney associated with a severe ureteral dilation. A new surgical approach and literature review

**DOI:** 10.1016/j.eucr.2025.102943

**Published:** 2025-01-13

**Authors:** Peranzoni Francesca, Birraux Jacques, Sommer Christelle, Sanchez Oliver

**Affiliations:** a Department of Pediatric Surgery, Centre Hospitalier Universitaire Vaudois, Lausanne, Switzerland; b Department of Pediatric Surgery, Hopitaux Universitaire de Genève, Geneva, Switzerland

**Keywords:** Crossed renal ectopy, Pediatric, Renal ectopy. laparoscopy, Retroperitoneoscopy

## Abstract

After horseshoe kidney, crossed renal ectopia (CRE) is the most common fusion anomaly of the kidney, with an incidence of 1:7000 autopsies. Most frequently the left kidney is the crossed ectopic component. In this article we present the first case of retroperitoneoscopic resection of a CRE and left ectopic multicystic dysplastic kidney with severely dilated ureter.

## 1 Introduction

After horseshoe kidney, crossed renal ectopia is the most common fusion anomaly of the kidney, with an incidence of 1:7000 autopsies. The fused variant it is 8 times more frequent than the non-fused variant. The true incidence of CRE is not known as it is predominantly a silent entity and in older adults, the fusion anomaly is usually discovered by coincidence.^1^ Most frequently, the left kidney is the crossed ectopic component.^2^

The vascularization of a cross kidney is always aberrant. The vascular supply can be provided by vessels generally arising from the lower aorta (near the bifurcation), the common iliac artery or the left external and internal iliac arteries.^3^ Due to numerous vascularization variants, radiological imaging is fundamental for studying the anatomical relationships with neighboring organs and vessels of a crossed ectopic kidney, allowing for the best surgical approach. 1

In a series of 36 patients most children with CRE presented within one year of age, with urinary tract infection being the most common presenting symptom. The most frequently associated anomaly was anorectal malformation.^4^

We present a case of left-to-right crossed and fused multicystic dysplastic ectopic kidney associated with left hydronephrosis and a severe left ureteral cyst. Our patient underwent a surgical resection of the left kidney and the dilated ureter via a retroperitoneal approach combined with cystoscopy and placement of a temporary JJ stent in the normal right ureter. This is the first case of laparoscopic retroperitoneal approach in a crossed ectopic and multicystic kidney described in the literature making this case report useful and interesting for the scientific community since the previously reported cases were not presenting with the same clinical features and were treated by laparotomy.^5^

## 2 Case report

A one-month-old child was referred to our hospital due to left a ectopic dysplastic kidney located beneath the inferior aspect of the right kidney, associated with a dilated left ureter and normal appearance of the bladder. He also presented with an undescended testis on the left side. The diagnosis was initially made by a routine antenatal ultrasound which also showed left cystic structure in the bladder described as a ureterocele and was confirmed by magnetic resonance imaging (MRI). Since the newborn period prophylactic antibiotics were started. A voiding cystourethrogram showed opacification of a short ureteral stump on the left. The bladder and the urethra had a normal appearance. No reflux was observed in the right kidney.

A hippuran dynamic renal scintigraphy showed a non-functioning left kidney and a right kidney with homogeneous parenchyma, compensatory function, and normal voiding capacity at one month of age. At the same age, blood tests revealed normal cystatin C levels at 1.65 mg/L, which was within the reference range. Creatinine was also normal at 25 μmol/L, and the electrolyte balance was within normal limits.

We initially opted for an observational approach, and the patient underwent serial ultrasonographic (USG) examinations twice per year during his first years of life, hoping for an involution of the dilated structures in the crossed left-to-right ectopic kidney. Routine visits to our outpatient clinic were scheduled to discuss the ultrasonographic results with the family and to ensure there were no breakthrough urinary tract infections. At almost one year of age, the left testis was still undescended, and surgical fixation was planned for 18 months of age. The left orchidopexy was performed through inguinal and scrotal incisions without complications in an ambulatory day hospital setting. During the operation, an absence of the left vas deferens was observed. This finding can be associated with alterations in the development of the mesonephric structures and the ureteric bud.

**Fig. 1:**
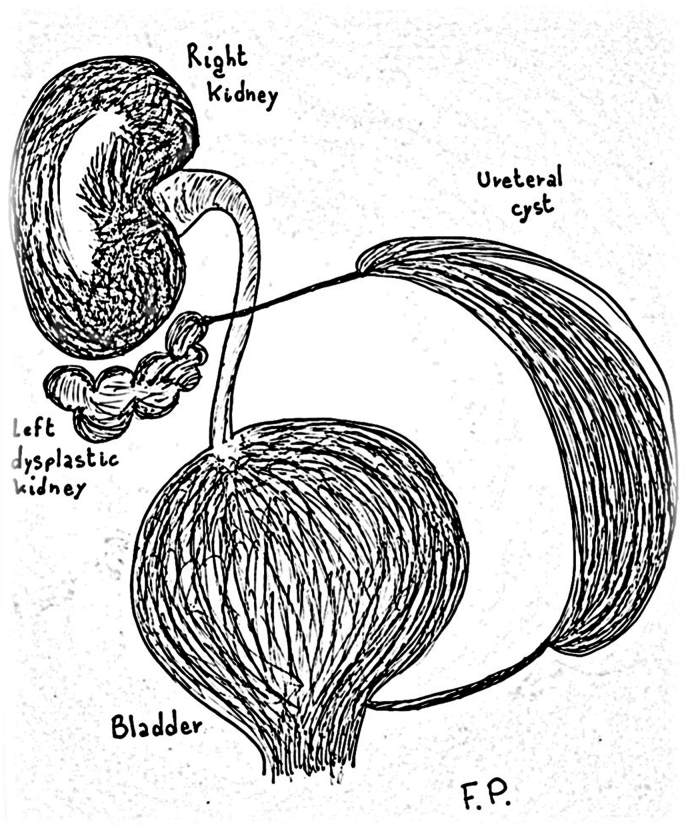
Anatomy simplified.

When the patient was almost three years old, the ultrasonographic report showed a worsening of the dilation in the left ureter, measuring 10 x 5 × 6 cm. Moreover, the ultrasound revealed the presence of sediment in the lowest part of the dilated structure. The right kidney was well-differentiated and vascularized, with dimensions of 88 × 36 mm (Fig. 1).

**Fig. 2:**
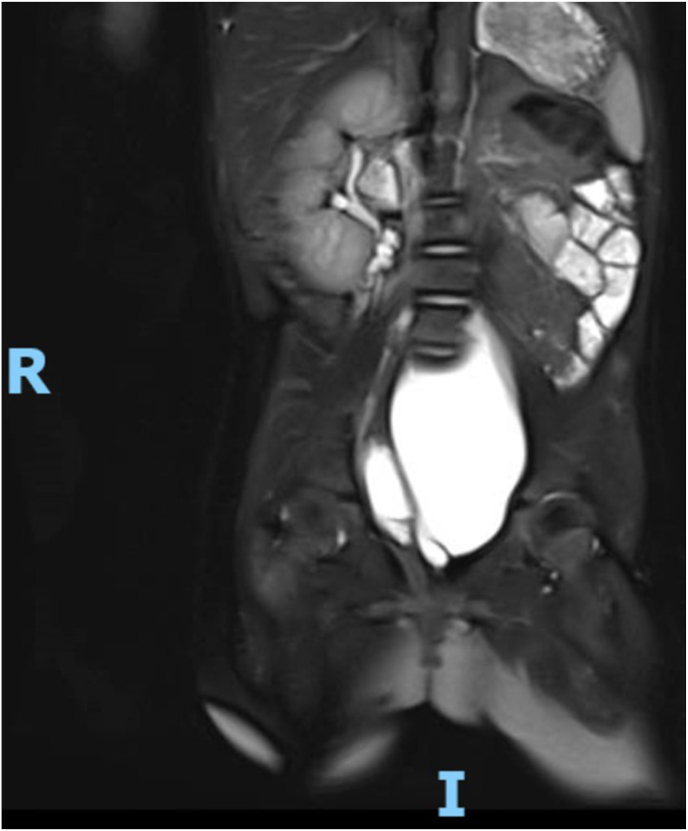
Right kidney, empty left kidney area.

**Fig. 3:**
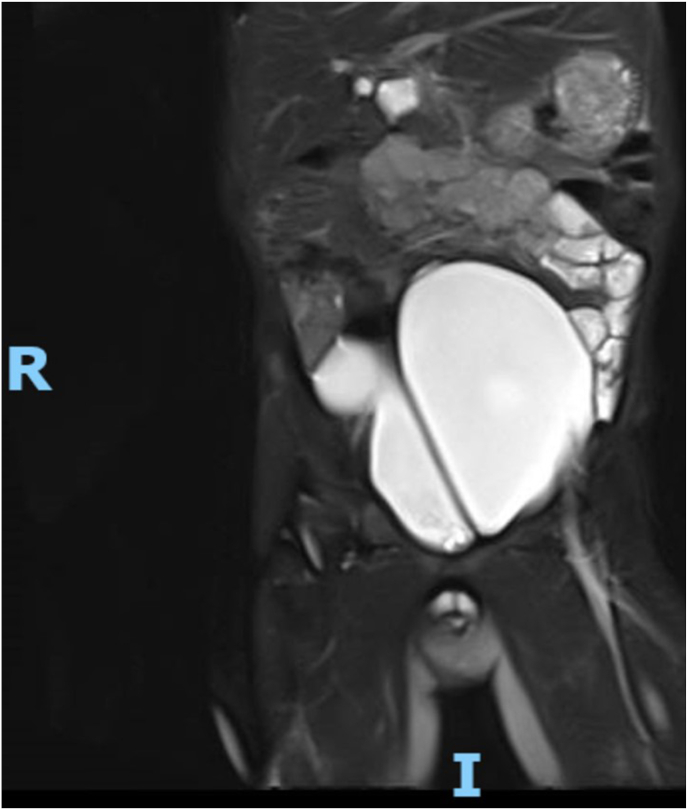
Left kidney dilated ureter.

A new MRI confirmed the findings and clarified the anatomy of the region (Fig. 2, Fig. 3).

Based on the worsening dilation and its effect on the bladder, a nephrectomy via a retroperitoneal approach was discussed with the family, who gave their consent for the operation.

The patient was 4 years old at the time of the operation. We began with a cystoscopy using a Wolf 8/9.8 cystoscope, which confirmed a normal urethra and a bladder deflected to the patient's right side. The right ureteral orifice was easily identified, but no left ureteral orifice was found. We placed a 4.7 French double J catheter in the right ureter to identify and protect the structure during the operation, leaving a traction stitch for removal. A urethral catheter was also inserted.

**Fig. 4:**
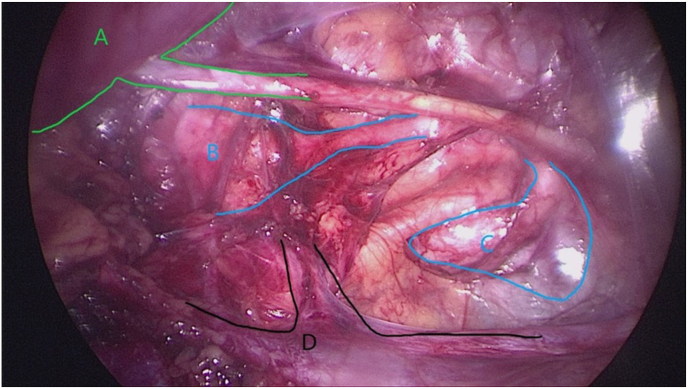
(A) Right kidney with JJ-stent in ureter, (B) left non-functioning crossed kidney, (C) dilated distal ureter, (D) vena cava and left kidney vessels.

We then performed a retroperitoneoscopic (Fig. 4) nephrectomy and partial left ureteral resection, placing the patient on left lateral decubitus. The first incision was made at the right renal angle. Then, a 5 mm trocar was placed and fixed to the wall using the Gaur technique, by the creation of a working space with a gloved finger inflated with 180 ml of air.^6^Under visual control, two additional 5 mm trocars were inserted above the right iliac crest and in the right flank. We began with the dissection of the pedicle of the left dysplastic kidney, which was sealed and divided with LigaSure®. We then gradually released the kidney. The large ureteral cavity was gradually freed beyond the midline. During dissection, the left ureteral cystic structure partially emptied, releasing a greenish cloudy liquid that was aspirated and washed away. We introduced a 5mm endoscope into the ureteral cavity and injected methylene blue through the bladder catheter placed at the beginning of the procedure. No reflux of liquid into the cystic structure was observed after filling the bladder with 180 ml. The cystic structure was incised, leaving its most distal part near the bladder in place. The left kidney and ureter were sent for anatomopathological examination. The double J stent in the right ureter was removed at the end of the operation using the traction stitch through the urethra, and a urethral catheter was positioned.

The patient was discharged without complications on the first postoperative day. For the first 5 days after the operation, he received intravenous antibiotic therapy with Ceftriaxone at a dose of 50 mg/kg once per day.

**Fig. 5:**
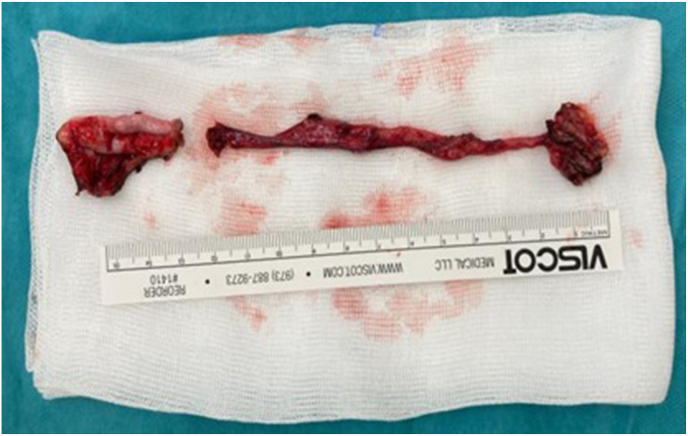
The left dysplastic kidney and ureter.

Histological examination of the specimen (Fig. 5) revealed fibrosed dysplastic renal parenchyma; no lesions were observed in the ureter.

One month after the surgery, the ultrasound showed compensatory hypertrophy of the right kidney, measuring 98mm on its largest axis, with a homogeneous structure and good corticomedullary differentiation. No pyelocalyceal dilation was noted. Near the bladder, a persistent ureteral structure measuring 3.7 x 2.3 × 3.5 cm was described.

At the latest clinic visit, two years after the surgery, the patient underwent clinical examination and ultrasonography. The ultrasound confirmed compensatory hypertrophy with no pyelocalyceal dilation of the right solitary kidney. A 4 cm × 3 cm x 2 cm residual cystic left paravesical structure was observed. Most importantly, the patient had no physical complaints and no urinary infections or bothersome urinary symptoms such as incontinence or dysuria. The surgical scars were small and discreet.

## 3 Discussion

Although the embryologic development of CRE has not yet been fully understood, many theories have been proposed to explain this condition. In 1952, Potter suggested that the ureteric bud on one side of the cloaca wanders over to the other side of the body and induces the tissue of the opposite metanephros to form additional kidney tissue.^7^ Another theory suggests that the ureteric bud induces the formation of a kidney in the metanephros on its own side, but the developing kidney is forced across the body by the pressure of the umbilical arteries.^8^ In 1960, some authors proposed another hypothesis, involving a positive attraction by the metanephros for both ureters in the absence of metanephric structures on the other side. This suggestion retains the principle of positive attracting forces within the embryo.^9^ Crossed renal ectopia (CRE) has been reported to occur two to three times more commonly on the right side than on the left, with the left kidney being the crossed ectopic component in a ratio of three right to two left. Male infants have a higher incidence of the condition, with a ratio of three males to two females.^2^

In accordance with previous reports, our patient was male, with a left-to-right fused ectopic kidney. The diagnosis of an ectopic kidney was possible through routine USG examination in the prenatal period. For confirmation of the diagnosis, MRI was necessary, and other complementary investigations were conducted, such as hippuran scintigraphy and voiding cystourethrography. It is also possible to use computed tomography, retrograde pyelography, and angiography to better determine the anatomy. Additionally, it is important to rule out other concomitant anomalies, such as urinary reflux and urethral valves, as well as non-urinary anomalies such as skeletal, gastrointestinal, genital abnormalities 10 and cardiopulmonary anomalies.

In our case, scintigraphy was useful in demonstrating the function of the orthotopic kidney and the lack of function in the dysplastic left kidney.

In the scientific literature, not many approaches to the surgical resection of ectopic kidneys are proposed. As stated above, only the laparotomy approach is mentioned for treating patients with a crossed ectopic multicystic dysplastic kidney with a ureterocele.^5^

## 4 Conclusion

In the current literature, this is the only reported case of a patient with left-to-right CRE involving a dysplastic kidney and ureteral dilation and cyst who underwent surgical resection of the ectopic kidney through a combined cystoscopy and laparoscopic retroperitoneal approach. This case report provides details of a unique presentation of CRE and outlines the steps for subsequent examinations, including imaging and laboratory studies, as well as treatment. Finally, we propose a new option for treating CRE through a laparoscopic retroperitoneal approach.

## CRediT authorship contribution statement

**Peranzoni Francesca:** Writing – original draft, Data curation. **Birraux Jacques:** Visualization, Validation, Supervision, Investigation. **Sommer Christelle:** Validation, Supervision, Methodology, Investigation, Conceptualization. **Sanchez Oliver:** Writing – review & editing, Visualization, Validation, Supervision, Methodology, Investigation, Conceptualization.

## Funding statement

This research did not receive any specific grant from funding agencies in the public, commercial, or not-for-profit sectors.

## Declaration of competing interest

None.
